# Study on the role of SLC14A1 gene in biochemical recurrence of prostate cancer

**DOI:** 10.1038/s41598-022-20775-7

**Published:** 2022-10-18

**Authors:** Bin Ye, Ke Ding, KaiXuan Li, Quan Zhu

**Affiliations:** 1 Department of Anesthesiology, Second Xiangya Hospital, Central South University, Changsha, 410008 Hunan China; 2 Department of Urology, Xiangya Hospital, Central South University, Changsha, 410008 Hunan China; 3 Department of Cardiac Surgery, Xiangya Hospital, Central South University, Changsha, 410008 Hunan China; 4 Department of Thoracic Surgery, Xiangya Hospital, Central South University, Changsha, 410008 Hunan China

**Keywords:** Prostate, Cancer, Surgical oncology

## Abstract

Prostate cancer (PCa) is a common malignant disease among men and biochemical recurrence (BCR) is considered to be a decisive risk factor for clinical recurrence and PCa metastasis. Clarifying the genes related to BCR and its possible pathways is vital for providing diagnosis and treatment methods to delay the progress of BCR. An analysis of data concerning PCa from previous datasets of The Cancer Genome Atlas (TCGA) and the Gene Expression Omnibus (GEO) was performed. Immunohistochemical (IHC) staining were used to evaluate the expression of SLC14A1 in prostate tissues. Kaplan–Meier analysis, Pearson correlation, and single sample Gene Set Enrichment Analysis (ssGSEA) were used to identify the potential pathway and molecular mechanism of the function of SLC14A1 in BCR of PCa. The expression of SLC14A1 is significantly reduced in prostate cancer cells and tissue comparing to normal prostate epithelial cell and para-cancerous tissue. As indicated by Kaplan–Meier analysis, High expression of SLC14A1 could increase the BCR-free survival time of PCa patients. This effect might be related to the interaction with miRNAs (has-miR-508, has-mir-514a2, and has-mir-449a) and the infiltration of B cells. SLC14A1 is a novel important gene associated with BCR of PCa, and further studies of its molecular mechanism may delay the progress of BCR.

## Introduction

Prostate cancer (PCa) is a common malignant disease and causes second-ranked cancer-related death among men^1^. The biochemical recurrence (BCR) of PCa is an important indicator of the prognosis of patients, and it often indicates the recurrence of the tumor. According to the guidelines of the European Association of Urology and the American Urological Association, BCR is defined as consecutive prostate-specific antigen (PSA) values no less than 0.2 ng/mL following radical prostatectomy (RP)^2,3^. It is worth pointing out that BCR does not mean clinical recurrence, but it is a decisive risk factor for PCa-specific mortality and overall mortality^4^. As the disease progressed without a second treatment after the diagnosis of BCR, 30% of patients have the median survival period of 5–8 years and around 32–45% among these patients would suffer PCa-specific mortality within 15 years^5^. Also, the clinically localized PCa, which is a significant cancer-specific survival benefit in patients after RP, still has nearly 30% PCa patients progressing into BCR after surgery^6,7^.

Similar to other types of tumors, the molecular mechanism of PCa development and patient prognosis is still unclear. Therefore, digging out the related genes of BCR in PCa can be beneficial to disease monitoring and treatment after RP. The human solute carrier family 14 member 1 (SLC14A1) gene, which is located on chromosome 18q12.1–21.1, contains about 30 kb nucleotides^8^. It is a solute carrier family gene that attracted people's attention by genome wide association studies (GWAS) and was considered to be related to the field of urothelial cancer^9^. The recent studies only indicate that SLC14A1 expresses differently between PCa and benign prostate tissue. SLC14A1 was found down-regulated in PCa tissue at around 2.88 fold and the castration will increase the expression of SLC14A1 by 3.05 fold, indicating that the expression of SLC14A1 gene in the prostate could be regulated by androgen, but the specific relationship of SLC14A1 and PCa has not been reported previously^10^.

In our study, we aimed to: (I) identify the relationship of SLC14A1 and PCa and verify the correlation between SLC14A1 and BCR using data obtained from the Gene Expression Omnibus (GEO) and The Cancer Genome Atlas (TCGA) database; (II) predict the correlation between SLC14A1 and miRNAs, and find the miRNAs which are related to BCR using Pearson correlation analysis and Kaplan–Meier survival curves; (III) analyze the potential biological signaling pathway of PCa, which SLC14A1 might involves in, by Gene Set Enrichment Analysis (GSEA) and GO and KEGG Pathway Enrichment Analysis.

## Materials and methods

### Data processing

The GEO database (https://www.ncbi.nlm.nih.gov/geo/) is a free public database and provides microarray and next-generation sequencing for users. Five gene expression profiles (GSE32448, GSE46602, GSE69223, GSE70768, GSE54460) were obtained from GEO database. The array data of GSE32448 contained 40 normal samples and 40 PCa samples^11^. GSE46602 included 14 normal samples and 40 tumor tissue samples^12^. GSE69223 consisted of 15 normal tissue samples and 15 PCa tissue samples^13^. GSE70768 included 74 normal prostate tissue samples and 125 PCa tissue samples^14^. GSE54460 was used for verification^15^. And it is worth noting that the normal prostate tissue includes not only the traditional normal prostate tissue but also the paracancerous tissue. The Transcripts Per Million (TMP) of TCGA and GTEx was downloaded from UCSC xena (https://xenabrowser.net/datapages/), and it was processed uniformly by the TOIL process, free of computational batch effects. TCGA-PRAD (The Cancer Genome Atlas Prostate Adenocarcinoma) clinical information counts and FPKM expression matrix are all downloaded from UCSC Xena (https://xenabrowser.net/datapages/). The TCGA and GEO datasets were extracted from a public database and required no ethical approval. The study was conducted by the Declaration of Helsinki (as revised in 2013).

### Determination and verification of the cut-off value of SLC14A1 gene expression

A total of 429 samples with BCR follow-up time in TCGA (experimental group) were included in the study, and 106 samples with BCR follow-up time in GEO (validation group) were included in the study. The cut-off value SLC14A1 gene expression was calculated by “surv_cutpoint” using the “survminer” package. In the experimental group, patients were be divided into 2 groups based on their SLC14A1 gene expression, which are high expression group and low expression group. Differential analysis was performed using the “edgeR” package. The same cut-off value was also used in the validation group for verification. Kaplan–Meier analysis with log-rank test was used to assess the difference in BCR-free survival between the high expression group and low expression groups with the help of the “survival” package in both experimental group and validation group.

### Immunohistochemical staining

Tissue chip containing 50 prostate cancer patients (including cancer and paracancerous tissues) was purchased from Guangzhou Wo Zhao Biotechnology Co., Ltd. Immunohistochemical staining (IHC) was performed to evaluate the expression of SLC14A1 of the prostate cancer tissue comparing with the prostate paracancerous tissue. Briefly, slide mounted sections were brought to room temperature and dried for 30 min. Slides were then placed in an antigen retrieval solution, which contained 10 mM sodium citrate in water (pH 6.0). The sections were incubated overnight at 4 °C in SLC14A1(1:150, Invitrogen, California, USA) after being blocked with 3% H_2_O_2_. The primary antibody was washed off with PBS-T (3 ×, 10 min) and slides were subsequently incubated for 1 h in a biotinylated goat anti-rabbit IgG (1:200, Vector Laboratories) followed by rinses in PBS-T. And then the sections were incubated with biotinylated secondary antibody (1:200, CWBio, Beijing, China) for 30 min at room temperature, and development was achieved with 3,3′-diaminobenzidine.

### Differential analysis of miRNAs based after grouping

Differential analysis was performed based on the SLC14A1 gene expression using the “edgeR” package, all the miRNAs profiles were downloaded using the “TCGA biolinks” package. Statistically different miRNAs were screened out for correlation analysis (Pearson correlation) to assessed the difference in miRNA expression between tissues with low and high SLC14A1 expression. In addition, Kaplan–Meier analysis was performed, too. All included genes have FDR < 0.01.

### Functional enrichment analysis and gene set enrichment analysis

Functional enrichment analysis and gene set enrichment analysis was conducted to identify the biological processes, cellular components, and molecular functions of SLC14A1 related to BCR using the “clusterprofiler” package, based on TCGA differential analysis and their multiples of difference.

### Differences in clinical phenotypes

The clinical data were also obtained from GSE54460, the clinical phenotypes comparison between the high expression and low expression group was performed to identify the relationship of SLC14A1 and clinical phenotypes.

### Differences in immune cells after grouping

One of the biggest advantages of ssGSEA (single sample Gene Set Enrichment Analysis) to quantify immune cell infiltration is that researchers could customize and quantify the types of immune infiltrating cells with the help of gene markers. The information of 24 immune cells was obtained from the currently recognized and used most immune cell markers^16^. Identify the immune cells of TCGA-PRAD and GSE46602 with ssGSEA, and Wilcoxon test were performed to compare the differences between groups of immune cells in different groups. In addition, multivariate cox regression analysis was also performed to screen out independent risk factors for BCR events.

### Statistical analysis

All statistical analysis was conducted in R v. 3.6.1. Wilcoxon rank-sum test is used to compare differences between groups for SLC14A1 and immune cells. Univariate cox analysis was used for screening of immune cells which were related to BCR. Log_2_foldchange > 2 and *p* < 0.05 were used for miRNAs screening. |log_2_foldchange | > 1 and *p* < 0.05 was used for functional enrichment analysis. All *p* values < 0.05 were considered statistically significant.

### Ethical approval and consent to participate

This study has been reviewed by the ethics committee of Xiangya Hospital of Central South University, and the ethics number is 202201002.

## Results

**Figure 1:**
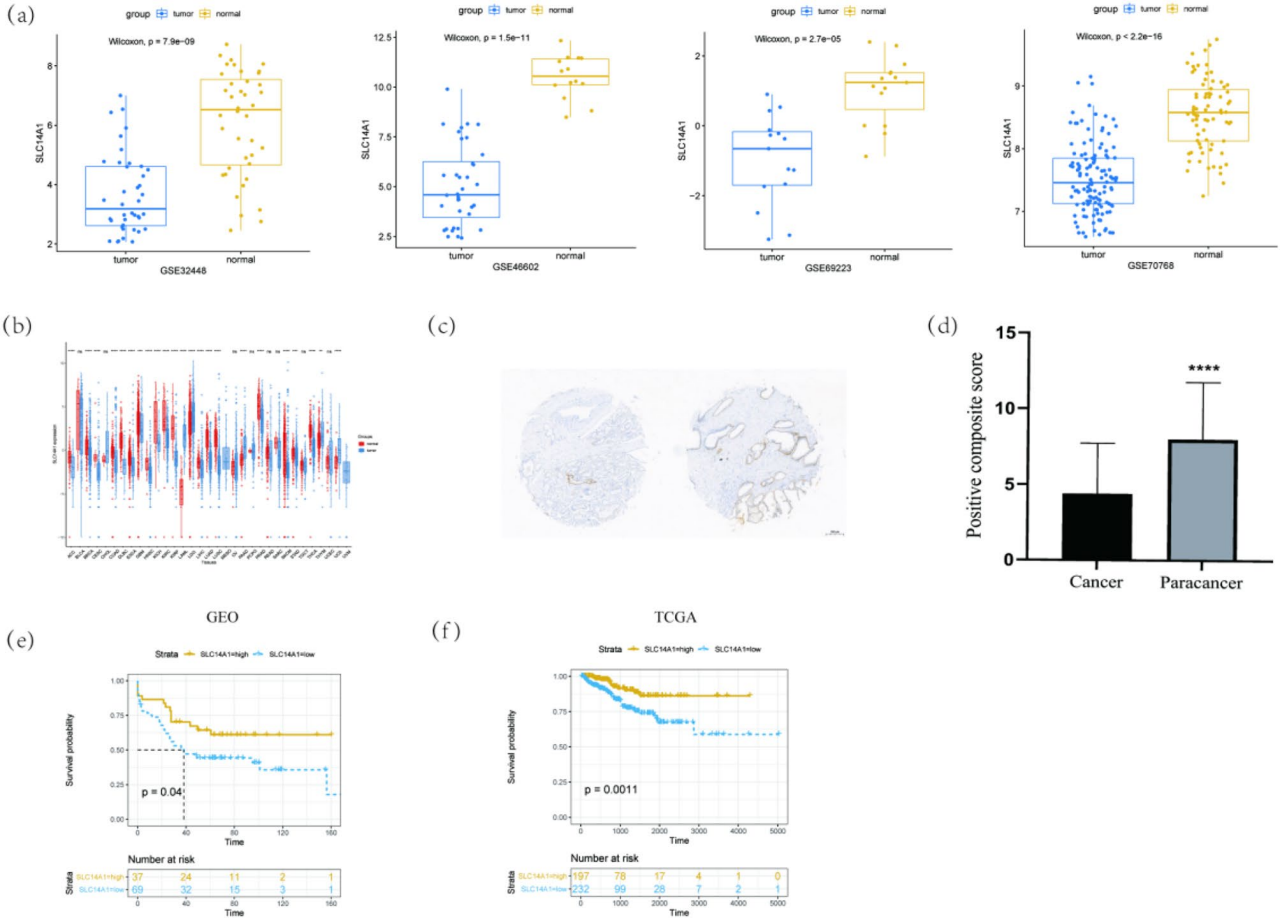
SLC14A1 gene is differentially expressed in PCa and other cancers and associated with PCa patients’ prognosis. (**a**) Comparison of SLC14A1 gene expression in PCa and normal tissue profiling datasets GSE32448, GSE46602, GSE69223, and GSE70768. (**b**) Comparison of the transcripts per million (TPM) value of SLC14A1 expression in other tumors and normal tissue. TPM values were obtained from TCGA datasets. (**c**) Representative micrographs of IHC staining for SLC14A1 in prostate cancer and paracancerous tissue. The scale bar is 200 μm. (**d**) Positive composite score of prostate cancer and paracancerous tissue. ****p < 0.0001 compared with cancer, n = 32. (**e**) Kaplan–Meier survival curves of experimental group, data were obtained from TCGA; (**f**) Kaplan–Meier survival curves of verification group, data were obtained from GEO.

Four expression profiles of SCL14A1 in normal and PCa tissue were obtained from GEO, and they were GSE32448, GSE46602, GSE69223, and GSE70768, respectively. The expression of SCL14A1 was significantly higher in normal prostate tissue compared to tumor tissue, the *p* values were 7.9^e−09^, 1.5^e−11^, 2.7^e−05^, and < 2.2^e−16^ respectively (Fig. 1a). When taking the expression of SLC14A1 in different cancer types and their related para-cencerous tissue into consideration (Fig. 1b). Among many cancer tissues and normal tissue samples, SCL14A1 expressed differently and was often lower in common tumors. In addition, we found that the expression of SLC14A was significantly reduced in PCa tissue via IHC staining in PCa tissue chip. The positive composite score (Assignment of positive IHC results score) in the prostate cancer tissue was significantly reduced compared to the paracancerous tissue, *p* < 0.0001 (Fig. 1c,d).

The clinical data of 429 patients were obtained from TCGA. According to the cut-off value of SLC14A1 gene expression, which was calculated by “surv_cutpoint” of survminer R package, the patients were divided into 2 groups. Additional 106 patients whose data were downloaded from GEO were enrolled in our study for external verification. Kaplan–Meier survival curves also showed that highly expressed SLC14A1 tends to have a lower recurrence rate of PCa (Fig. 1e). Internal verification showed that the high expression group got less incidence of BCR, which indicated that SLC14A1 was a protective factor for PCa recurrence (Fig. 1f).

A total of 429 samples with BCR follow-up time in TCGA (experimental group) were enrolled in our study, and Table 1 shows the differences between the high SLC14A1 expression group and the low expression group. The Gleason score of the high group was less than the low group (0.94 vs. 1.04, *p* < 0.001), which indicated that high expression of SLC14A1 might be a benefit of PCa prognosis. In addition, the low expression group had a much higher rate among stage pathologic N1 PCa compared with the high expression group (11.3 vs. 23.8, *p* = 0.002).

**Table 1:** Differences in clinical phenotypes after grouping.

Clinical features	high	low	*p*
n	197	232	
**Clinical_T (%)**			0.073
T1	77 (47.0)	76 (39.6)	
T2	72 (43.9)	82 (42.7)	
T3	15 (9.1)	33 (17.2)	
T4	0 (0.0)	1 (0.5)	
Gleason_score (mean (SD))	7.39 (0.94)	7.86 (1.04)	< 0.001*
**Laterality (%)**			0.76
Bilateral	175 (90.7)	202 (88.2)	
Left	7 (3.6)	10 (4.4)	
Right	11 (5.7)	17 (7.4)	
Pathologic_N = N1 (%)	19 (11.3)	49 (23.8)	0.002*
**Pathologic_T (%)**			0.009*
T2	85 (44.0)	70 (30.4)	
T3	106 (54.9)	154 (67.0)	
T4	2 (1.0)	6 (2.6)	
OS = 1 (%)	5 (2.5)	7 (3.0)	1
OS × time [mean (SD)]	1075.06 (737.22)	1194.52 (843.60)	0.122
BCR = 1 (%)	14 (7.1)	44 (19.0)	< 0.001*
BCR_time (mean (SD))	1012.31 (714.96)	1051.63 (824.54)	0.601

*Statistically significant (α = 0.05).

*SD* Standard Deviation, *N* Node, *T* Tumor, *OS* Overall Surviva, *BCR* Biochemical Recurrence.

**Figure 2:**
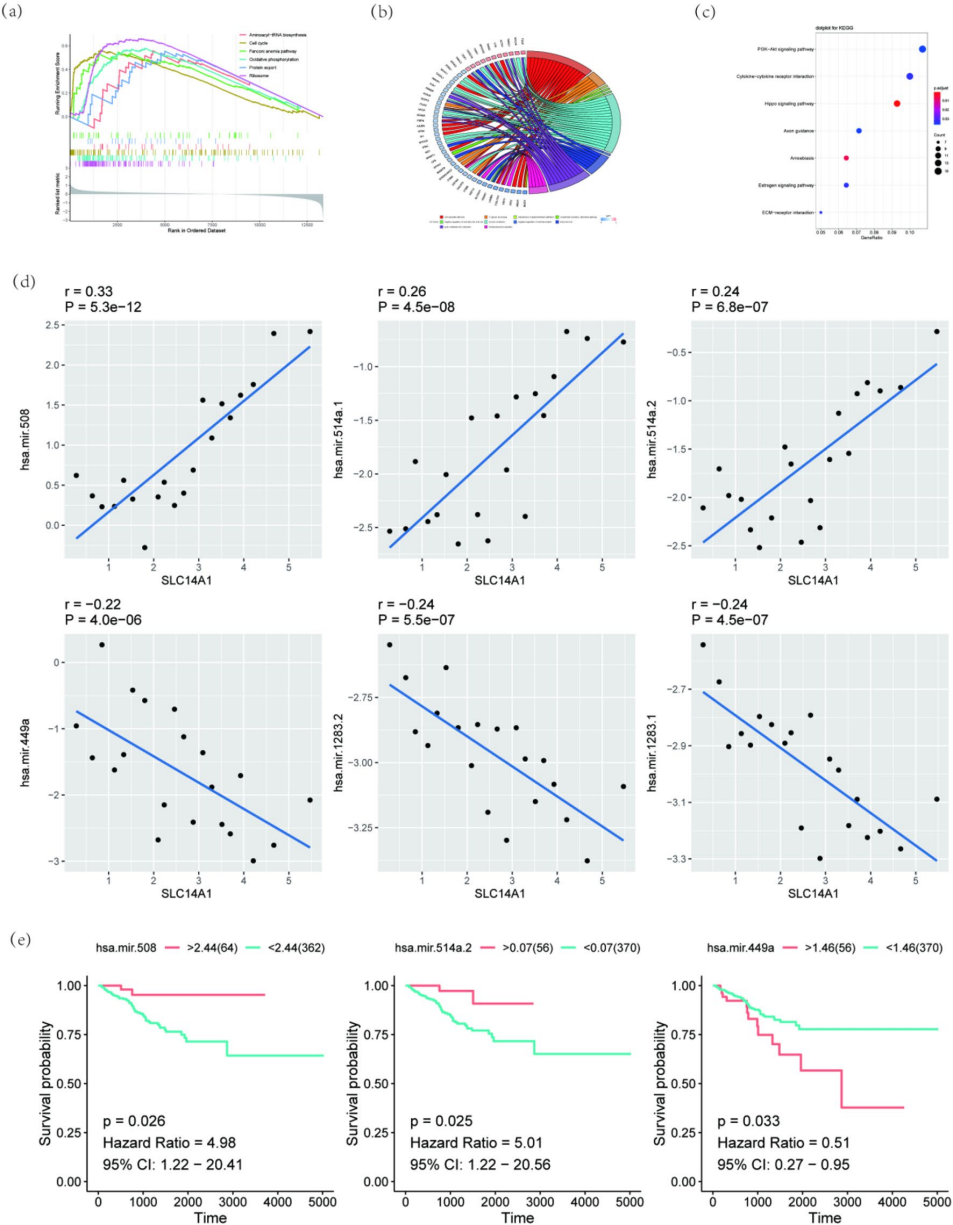
Functional analysis and miRNA association analysis of SLC14A1. (**a**) Gene Set Enrichment Analysis of high expression group and low expression group. (**b**) GO analysis, each strip represents a pathway. (**c**) KEGG analysis. Each small point in the graph corresponds to a pathway, and the color is sorted by *p* value from small to large according to red, orange, yellow, green, blue, indigo, and purple. The smaller the *p* value is, the more color tends to red, and the larger the point is, the more genes in the pathway are. (**d**) Correlation analysis between ± top 3 differential expression miRNAs and SLC14A1. (**e**) Kaplan–Meier survival curves of the ± top 3 differential expression miRNAs that had a statistical significance.

There were 6 biological pathways with the top enrichment score being selected by GSEA of the high expression group and low expression group (Fig. 2a). They were aminoacyl-tRNA biosynthesis, cell cycle, Fanconi anemia pathway, oxidative phosphorylation, protein export, and the ribosome.

In addition, the mRNAs from the comparison of the high expression group and low expression group were significantly involved in cell–substrate adhesion, O–glycan processing, and maintenance of gastrointestinal epithelium in GO. And the most different pathways in KEGG were the PI3K–Akt signaling pathway, cytokine–cytokine receptor interaction, and hippo signaling pathway (Fig. 2b,c)^17–19^.

The same cut-off point was used to distinguish the high expression group and the low expression group. A total of 22 miRNAs expressions were statistically different in the high expression group and the low expression group (log_2_foldchange > 2 and *p* < 0.05). And the ± top 3 miRNAs which were mostly related to SLC14A1 were selected by Pearson correlation (Fig. 2d). The 6 selected miRNAs were carried out to perform BCR-free Kaplan–Meier plots. Three miRNAs showed statistical differences comparing the high expression group and low expression group (Fig. 2e).

**Figure 3:**
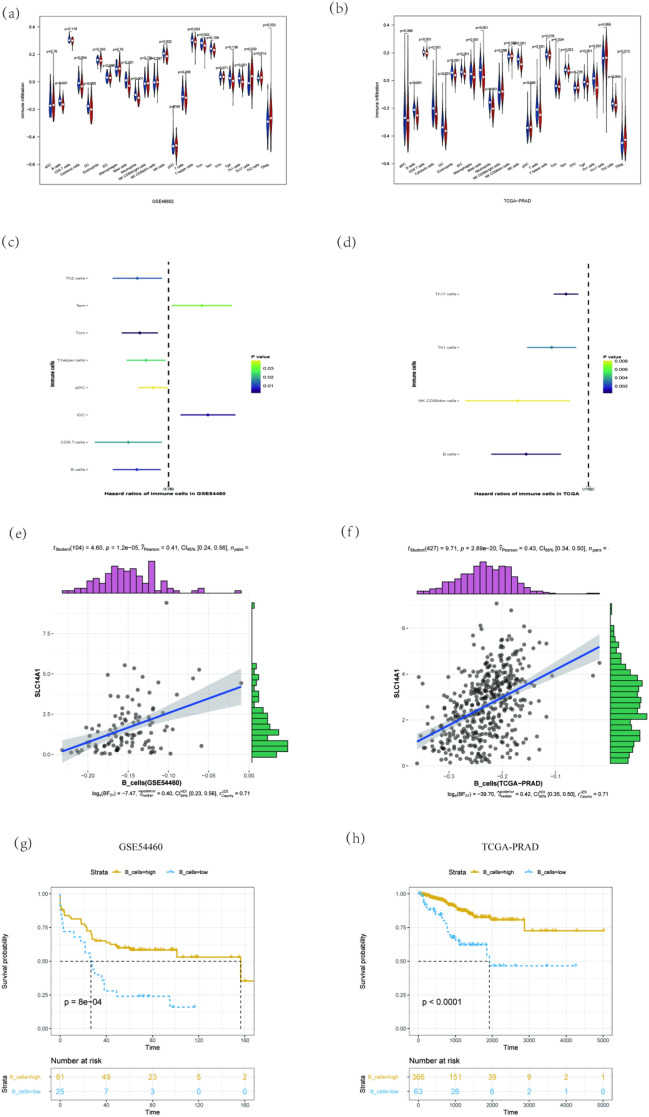
The associated analysis of SLC14A1 and immune cells. (**a**) and (**b**) used Wilcoxon test, (**c**) and (**d**) used univariate cox analysis. (**e**) and (**f**) show the correlation between SLC14Aa, data were obtained from GSE54460 and TCGA-PRAD, respectively. (**g**) and (**h**) show the Kaplan–Meier survival curves of B cells, data were also obtained from GSE54460 and TCGA-PRAD, respectively.

To identify the relationship between SLC14A1 and immune cells, differential analysis was performed using the Wilcoxon test. Violin illustration showed that B cells, cytotoxic cells, mast cells, neutrophils, NK cells, Tcm, Tem, Th1 cells, and T Reg were statistically different (Fig. 3a,b). Nevertheless, when taking them into univariate cox analysis, only B cells showed correlation with BCR events (Fig. 3c,d).

The correlation between SLC14A1 and B cells and Kaplan–Meier survival curves of B cells were shown in Fig. 3e–h. The Pearson correlation coefficient of the B cells and SLC14A1 were 0.41 and 0.43, respectively. Although the Pearson correlation coefficient only indicated that they were moderately related, Kaplan–Meier survival curves showed the significant statistic difference between B cells high group and B cells low group. To further validate independent risk factors for BCR events, multivariate cox regression analysis was performed (Table 2). Low SLC14A1 expression was more likely to cause BCR events, and B cells, pathologic_N, and gleason score were all independent risk factors for BCR events.

**Table 2:** Multivariable Cox regression of characteristics relating to BCR events.

Characteristics	*p*	HR value
SLC14A1_group (low vs. high)	0.025*	3.4 (1.2–10)
SLC14A1	0.024*	1.5 (1.1–2.3)
B_cells	0.035*	0.00084 (1.1e−06–0.61)
Pathologic_N	0.54	0.81 (0.42–1.6)
Pathologic_T	0.0026*	1.7 (1.2–2.5)
Gleason_score	0.023*	1.5 (1.1–2.1)
hsa.mir.508	0.99	1 (0.77–1.3)
hsa.mir.514a.2	0.15	0.83 (0.65–1.1)
hsa.mir.449a	0.63	0.97 (0.86–1.1)

*Statistically significant (α = 0.05).

*BCR* biochemical recurrence, *HR* hazard ratio.

## Discussion

The incidence of PCa in men is very high, and RP is an important treatment modality. However, BCR after RP needs to arouse people's attention, because it will reduce the survival time of PCa patients after surgery. It is important to figure out the occurrence of BCR, and early detection of BCR will promote the treatment efficacy, which might include targeted radiotherapy or surgery^20^. Our research found that SLC14A1 was associated with the BCR of PCa, although some genes, such as cripto-1 (CR-1), abnormal spindle microtubule assembly (ASPM), C-X-C motif chemokine ligand 12 (CXCL12), epithelial cell transforming sequence 2 (Ect2), a four-long non-coding RNA (lncRNA) signature (RP11-108P20.4, RP11-757G1.6, RP11-347I19.8, and LINC01123) and pleomorphic adenoma gene like-2 (PLAGL2), were indicated to be related to PCa prognosis^21–26^, the studies of SLC14A1 in PCa were limited.

SLC14A1 encodes type-B urea transporter (UT-B), which facilitates the rapid and passive cross-membrane movement of urea^27^. The low expression could induce urea accumulation which might influence the PCa cells. In the UT-B knock-out mice, the urea concentration reached about 9 times that of the wild type which caused severe apoptosis and DNA damage in the urothelial cells^28^. A high concentration of urea could cause cell cycle delay in the G2/M phase and G0/G1 phase, and lead to apoptosis and death of cells^29^. In addition, urea accumulation will alter arginine metabolism, thereby increasing the level of inducible NO synthase (iNOS) in cells^28^. Hypoxia-inducible factor-1 (HIF-1) is stabilized by a high concentration of nitric oxide (NO) catalyzed by iNOS, and HIF-1 is recognized to be related to tumors^30^. Therefore, in our study, the low expression of SLC14A1 seemed to be associated with BCR in PCa, the molecular mechanism might be multiple pathways induced by a high concentration of urea.

Some researchers found that when transfecting cancer cells with miR-508 mimics, the cancer cells significantly reduce their ability of cell proliferation, migration, and invasion. And when cancer cells were transfected with miR-508 inhibitor, the effect was opposite to the previous^31,32^. There is no clear report on the relationship between miR-514a2 and cancers. But as for miR-449a, has a recognized tumor suppressor effect, especially in PCa. Gupta et al. found that c-Myc, a key factor that promotes cell cycle regulation, will be downregulated with miR-449a upregulated^33^. Similarly, Noonan et al. found that miR-449a was a miRNA component in the Rb pathway, and its tumor suppressor-like effect partly depends on the Rb status in PCa cells^34^. So miR-449a might be a key factor in PCa cells cycle arrest and senescence, which corresponding to our GESA results. Also, loss of miR-449a would cause PrLZ overexpression and promoted PCa metastasis^35^. And Cumar et al. found that SIRT1, a multifaceted NAD^+^-dependent protein deacetylase, will upregulate with the loss of miR-449a and promoted the invasion of PCa cells^36^.

Studies have shown that enrichment-based pathway diagrams on androgen-regulated proteomics datasets revealed significant imbalances in aminoacyl-tRNA synthetase, indicating increased protein biosynthesis which was a sign of PCa progress^37^. Cell cycle, which is closely related to the proliferation of PCa cells. The loss of controlling of the cell cycle monitoring mechanism can also occur in any link of cell proliferation damage induction, DNA repair, cell death, etc., finally cause the cell's genome instability. In addition, the biallelic mutation of Fanconi Anemia (FA) will not only lead to the phenotype of FA but also cause the instability of the genome and additional mutations in somatic cells, which leads to the susceptibility of many different types of cancer. And FA mutated had been found in PCa^38^. And the other pathways which were enriched in the SLC14A1 high expression group, such as oxidative phosphorylation, and ribosome, were all important pathways in the development and recurrence of PCa^39–41^.

Gleason grading is currently a widely used method of histological grading of prostate adenocarcinoma. Because Gleason classification is well related to biological behavior and prognosis, it is gradually recognized and used more and more widely, becoming an important reference indicator for formulating PCa treatment plans. A smaller Gleason score often indicates a better prognosis. In our study, patients with high expression of SLC14A1 got a lower Gleason score, which promoted survival of PCa patients and can predict the incidence of BCR.

B cells mainly exist in the lymph fluid circulating in the lymphatic vessels and are an important cellular component of the body's immune response function. The release of CXCL 13 from tumor cells could lead to B cells infiltration, which was considered as an important role in PCa progression^42^. In the PCa mouse model, the presence of immunosuppressive B cell subsets is associated with the accelerated recurrence of castration-resistant PCa^43^. In the pathological section of PCa, a large amount of B cell infiltration is related to the failure of chemotherapy of PCa^43,44^. These findings suggested that SLC14A1 might promote PCa immunology and a SLC14A1-targeted therapy could be created to delay tumor progression using the potential interaction of SLC14A1 and B cells.

## Conclusion

Here, we identified a novel gene which related to the BCR of PCa and high expression of SLC14A1 could reduce the occurrence of BCR. This effect of SLC14A1 may be related to the interaction with miRNAs (has-miR-508, has-mir-514a2, and has-mir-449a) and the infiltration of B cells. However, further basic research and clinical trials are needed to further prove our point and determine the molecular pathway that how SLC14A1 can reduce BCR in prostate cancer.

## Data Availability

All our data is pulled from public databases. The datasets GSE32448, GSE46602, GSE69223, GSE70768 and GSE54460 used and analyzed in the current study are all from the GEO database(https://www.ncbi.nlm.nih.gov/geo/). The Transcripts Per Million (TMP) of TCGA and GTEx was downloaded from UCSC xena (https://xenabrowser.net/datapages/). TCGA-PRAD (The Cancer Genome Atlas Prostate Adenocarcinoma) clinical information counts and FPKM expression matrix are all downloaded from UCSC Xena (https://xenabrowser.net/datapages/).
